# How Does Adolescents’ Usage of Social Media Affect Their Dietary Satisfaction?

**DOI:** 10.3390/ijerph19063621

**Published:** 2022-03-18

**Authors:** Harry Jeong, Kwangsoo Shin

**Affiliations:** 1 Central Research Institute, Dr. Chung’s Food Co., Ltd., Cheongju 28446, Korea; harry@g.cbnu.ac.kr; 2 Graduate School of Public Health and Healthcare Management, Songeui Medical Campus, The Catholic University of Korea, Seoul 06691, Korea

**Keywords:** food consumption pattern, food consumer, adolescent health, adolescent food, convenience food, cost-effective consumption, sound eating, SEM, mediation

## Abstract

In order to improve the health status of adolescents, studies are needed to illuminate the essence of their general and dietary lifestyle. Thus, we conducted this study to verify meaningful relationships between adolescent usage of social media (USM), which plays an important role in their life, their food consumption behavior (FCB), and their dietary satisfaction. This study used two analysis methods: *t*-tests and structural equation modeling (SEM). This study verified whether there was a significant difference in adolescent FCB depending on their USM using *t*-tests. This study proposes that the following FCBs showed significant differences between users and non-users of social media in adolescents: a tendency to try new types of food (t = 2.134, *p* < 0.05), a tendency to avoid foods with harmful risks such as suspected spoilage (t = 3.513, *p* < 0.001), a tendency to eat bread or fruit for a simple breakfast (t = −3.893, *p* < 0.001), and a tendency to often use home meal replacements (HMR), eat out or have food delivered (t = −3.245, *p* < 0.01). Furthermore, this study used SEM to verify the causal relationship between adolescent USM and their dietary satisfaction. According to the results of SEM, adolescents’ USM mediated by the FCB of preferring convenience fully mediates the negative relationship between adolescent USM and their dietary satisfaction (*p* < 0.01). It is necessary to reverse the situation in which adolescent dietary satisfaction decreases as their FCB of preferring convenience increases. Government regulations for food companies and autonomous efforts for quality improvements on their part are needed.

## 1. Introduction

The risk for chronic diseases such as hypertension, diabetes, and chronic kidney disease is increasing over time [1]. During the COVID-19 pandemic, these chronic diseases are even more prevalent [2]. This has become a serious problem that threatens everyone, regardless of age. Managing one’s diet is a very important factor in preventing or treating chronic diseases. A healthy diet is necessary from adolescence. Nevertheless, it is not easy to maintain a healthy diet for adolescents [3]. The purpose of this study is to investigate the effect of adolescents’ USM on their dietary lifestyle. In addition, the ultimate aim of this study is to present insights to help improve adolescents’ dietary satisfaction.

Previous studies have been limited to investigating the influence of adolescents’ USM on their eating behavior. There are some studies on how work affects adolescents’ dietary lifestyle [4]. Adolescents’ USM was established in the study as an integral factor affecting their health. Most adolescents share food content that they are interested in freely using social media and respond to content shared by other users. Holmberg et al. [5] found that 85% of adolescent users shared images featuring food items. Serenko et al. [6] argued that adolescent USM affected their sleep duration and healthy eating habits. Albert [7] reported that the USM of adolescents was related to their consumption of fast food and junk food. Chung et al. [4] studied adolescent peer influence on eating behaviors via social media.

FCB is influenced by demographic and social factors such as gender [8], age [9], and family size [10]. FCBs are often understood to have a social meaning, in addition to providing nutrients to the body [11]. In this context, sharing food as an individual-level FCB could have the effect of reducing food waste at the consumer level, along with potential positive environmental and economic effects. It has been found that food sharing has a positive effect on interpersonal relationships [12]. Whether adolescents use social media or not will have meaningful effects on the food consumption of those who form relationships through social media.

The research question of this study is the following: how does adolescents’ USM affect their dietary satisfaction? Thus, this study examines whether there is a difference in the FCB of adolescents depending on their USM and examines the relation between USM and dietary satisfaction mediated by FCB. This study was based on a 2020 consumer behavior survey on food targeting adolescents (n = 622), which was conducted by the Korea Rural Economic Institute (KREI), a government research institute in Korea. The survey provides data on the subjects’ FCB.

This study contributes to filling the gap in the literature on the relationship between adolescents’ USM, their FCB and their dietary lifestyle. This study can be referred to for food consumption education on the subject of adolescents. Guidelines for behaviors to increase adolescents’ dietary satisfaction can be established in reference to this work. It can also give food companies the insights they need for considering the health of adolescents.

The remainder of this study is organized as follows. The study starts with a literature review, covering adolescents’ USM and their FCB, as well as their dietary satisfaction. The subsequent section presents the methodology applied in this study, with a description of the dataset employed. Section 4 presents the results, while Section 5 and the final section offer a discussion and conclusions.

## 2. Theoretical Background and Hypothesis

### 2.1. Social Media and the Dietary Life of Adolescents

Adolescents are defined as young people between the ages of 10 and 19 [13]. Adolescence is a transition period of growth and development from childhood to adulthood [14]. During this phase, adolescents usually establish behavioral patterns related to diet, physical activity, substance use and sexual activity that can protect their health [13]. Adolescent resources are fundamental to later life health and determine trajectories into the next generation [15]. In particular, the lifestyle and FCB established during this phase are important factors directly affecting health throughout the lifespan.

Social media refers to communication tools based on the internet for sharing ideas, information, images, and other content [16]. It includes Facebook, Twitter, YouTube etc. Since social media can be accessed in real time using a smartphone and information can be easily shared, the dependence on social media is increasing in the lives of adults as well as adolescents [17]. Hur and Gupta [18] report that about 85% of adolescents between the ages of 12 and 17 use social media. Social media is an inseparable part of adolescent life [19]. Hence, we cannot ignore adolescents’ USM when we study their dietary life.

Previous studies on adolescents’ USM and their dietary life so far have mainly focused on sleep time and types of meals. Wojdan et al. [20] argued that young people’s USM had a negative effect on schooling and sleep time. In a study of the impact of adolescents’ USM on health-related outcomes in the UK, Serenko et al. [6] reported that USM mediates reduced sleep duration and decreased healthy eating. Albert [7] found that, in a sample of predominantly Latino middle school students, USM and other entertainment media use was negatively correlated with consumption of fruit and vegetables. In addition, it was found that they were strongly correlated with the consumption of fast food and junk food.

### 2.2. Social Media Goals

As one method of overcoming the problem of information asymmetry between product provider and consumer, consumers use social media [21]. Content providers on social media can be categorized as: company, government, and user communities. In terms of the consumption of products, companies provide basic information about products, and user communities respond to them and share various experiences with current users and future users. In this process, influencers also play a role, and sometimes exert more influence on future users. Different kinds of social connections have been formed between influencers and users. We can also call the users followers. The social connections between influencers and followers affect the followers’ purchase intentions [22]. In the case of adolescent followers, this trend is even more pronounced. Therefore, the government needs to provide comprehensive information across product categories through public service channels.

Consumers can process available information and make more conscious and rational decisions in a thoughtful manner [23]. The ultimate purpose of consumer decision-making, whether it is instinctive or learned, is to pursue happiness. Hoffman and Novak [24] proposed a new theoretical framework for social media goal pursuit. They found a clear link between social media goal pursuits and subjective well-being. It was found that the users who pursued interpersonal interactions were happy, and that the content creators who pursued content interaction were also happy.

### 2.3. Dietary Satisfaction

Dietary satisfaction is related to satisfaction with the quantity and quality of food, the planning and preparing of meals, and the acceptability of eating behaviors [25,26]. It is important to purchase and cook the proper raw materials, and to eat food that suits one’s preference. In fact, various factors are considered when choosing food. There are objective factors such as food safety, food temperature, menu variety or food presentation, and subjective factors such as taste and the service satisfaction of providers [27]. Besides these factors, the attitude towards food and dietary habits also influences dietary satisfaction. Adachi [28] found that eating habits, such as having a regular breakfast and eating a variety of foods, were important factors affecting dietary satisfaction.

### 2.4. Hypotheses

FCBs vary with factors such as age, income, and level of education [29,30]. In particular, differences in lifestyle depending on age have been revealed [31]. Dietary lifestyles also depend on age [32]. Compared to adults, adolescents enjoy relatively new challenges and are more sensitive to trends. Adults take a more conservative approach to food than younger people. Dietary lifestyle or FCBs could be categorized into 4 groups, depending on the factors consumers focus on; convenience, gourmet, health, and economic factors [33,34,35]. Considering the importance of USM in adolescent life, this study proposes the following hypothesis.

**Hypothesis** **1** **(H1).** *Adolescent USM affects their FCBs.*

Modern people usually consume with considerations of health in mind. They count calories when taking in nutrients [36,37]. The types of food for which food safety is not guaranteed are also subject to regulations [38,39]. It is the responsibility of food companies to guarantee the safety of food, and customers should be able to complain about products of course. Customers will prioritize food safety in valuing their health [40]. As this FCB affects the dietary lifestyle of adolescents, this study proposes the following hypothesis:

**Hypothesis** **2** **(H2).** *Adolescents’ USM mediated by the FCB of valuing their health increases their dietary satisfaction*.

Individuals generally have a tendency to purchase products with the best quality and a low price [41]. There has been an increasing tendency to value cost-effectiveness recently [42]. One of the marketing strategies of companies responding to customers who want to achieve cost-effectiveness is to emphasize the certification of their product. Certifications such as HACCP or GAP are universally recognized as having a relatively good quality in the food industry [43]. As this FCB affects the dietary lifestyle of adolescents, this study proposes the following hypothesis:

**Hypothesis** **3** **(H3).** *Adolescents’ USM mediated by the FCB of valuing cost-effective consumption increases dietary satisfaction*.

Sound eating habits are the key practice for keeping the body in balance [44,45]. It is beneficial not to be a picky eater, but to instead eat a variety of diets and to try new foods [46]. Eating delicious food makes people happier. Eating regularly is important for maintaining the proper signaling system in the human body [47]. Harmful risks should be closely observed to prevent them from entering the body through food [48]. As this FCB affects the dietary lifestyle of adolescents, this study proposes the following hypothesis:

**Hypothesis** **4** **(H4).** *Adolescents’ USM mediated by the FCB of keeping sound eating habits increases dietary satisfaction*.

The consumption desire for convenience is increasing [49,50]. The tendency to eat simple breakfasts has risen [51]. The rate of consumption of packaged foods or pre-processed agricultural products for lunch or dinner is also increasing [52,53]. With the development of technology, a variety of HMRs are supporting the trend for convenience [54].

**Hypothesis** **5** **(H5).** *Adolescents’ USM mediated by the FCB of preferring convenience increases dietary satisfaction.*

**Figure 1:**
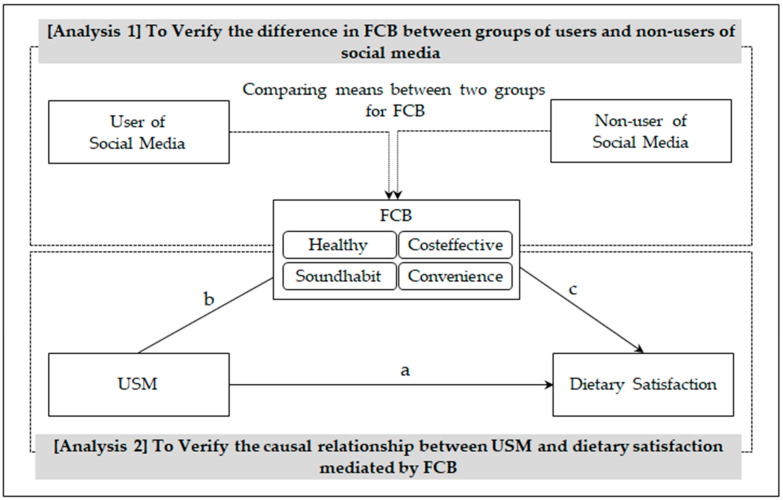
Overall research outline. Note: Healthy refers to the FCB of considering a consumer’s health; Costeffective refers to the FCB of valuing cost-effective consumption; Soundhabit refers to the FCB of keeping sound eating habits; Convenience refers to the FCB of preferring convenience; “a” refers to a direct path from USM to Dietary Satisfaction, “b” and “c” are indirect paths from USM to Dietary Satisfaction.

This study established hypotheses on the correlation between the USM, the FCB and the dietary satisfaction of adolescents. Two analyses are conducted to verify the hypotheses. For hypothesis 1, this study compared means of FCBs between users and non-users of social media. For hypotheses 2 to 4, this study conducted SEM to verify the causal relationship between USM and dietary satisfaction. Figure 1 shows the overall outline of the research aims.

## 3. Methodology

### 3.1. Research Procedure

The ‘Consumer behavior survey for foods’, which is a national survey approved by the Korean government, was conducted by the KREI. It shows the demographic characteristics and FCBs of young and adult consumers. Respondents of this survey were investigated regarding overall issues related to their FCBs, nutrition, and health. We extracted data related to USM, FCB, and dietary satisfaction for adolescent respondents only.

Before presenting the analysis, we explain how data were collected and variables were defined. Then, we conducted a factor analysis for removing variables that are not included in the factor or have a low importance even if they are included. The subsequent procedure, a reliability analysis, was performed to confirm whether the items within the factors were consistent.

Two analyses were conducted as part of this study. First, an independent sample *t*-test was conducted to verify differences of FCB between adolescent groups of users and non-users of social media. Second, SEM was conducted to prove the causal relationship between USM and dietary satisfaction mediated by FCB.

**Figure 2:**
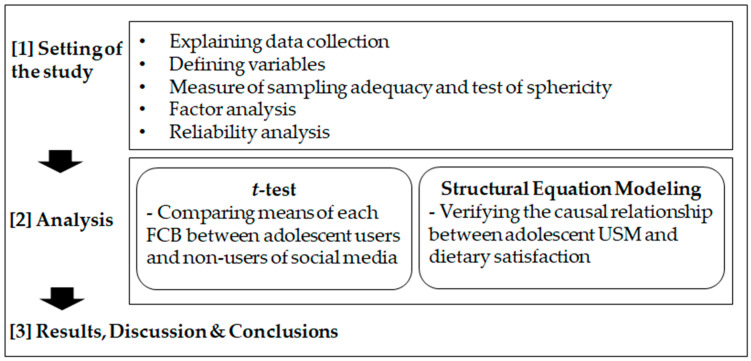
Research procedure of the study.

The research procedure is summarized in Figure 2.

### 3.2. Data

The survey by the KREI was conducted on the household level and the household member level, including adolescents, from June to August 2020. The sample was designed based on 3300 households from the 2019 survey and 690 randomized households from Statistics Korea. The KREI sent a survey questionnaire to 3990 households, and 622 adolescents responded. The respondent rate was 15.6%. Since the survey was for the overall level of household members, the non-response rate of the households consisting of only adults was high. Participants could select one of the following survey methods: a face-to-face interview survey using computer-assisted personal interviewing (CAPI), a self-administered survey, or an online survey.

### 3.3. Participants

There were 622 respondents (boys, 52.1%; girls, 47.9%). The mean age of participants was 16.1 ± 1.5 years (boys, 16.5 ± 1.6; girls, 16.1 ± 1.5 years).

### 3.4. Variables

USM was used as an explanatory variable in both analysis 1 and analysis 2. Each FCB was treated as a dependent variables in analysis 1, and as a mediating variable in analysis 2. Dietary satisfaction was used as a dependent variable in analysis 2. The definitions of the variables are summarized in Table 1. Respondents were to rate each item on a 5-point Likert scale, from 1 (absolutely disagree) to 5 (absolutely agree).

### 3.5. Measurement

#### 3.5.1. FCB

Factor analysis was performed to understand how sub-factors are classified for FCB. Related variables were grouped together to form factors, and they had mutually independent characteristics; accordingly, the characteristics of variables could be known. As a factor extraction method, principal factor analysis was conducted, and four factors were extracted by promax rotation. As a result, the following items were excluded from the analysis because they impair validity: buy_cert, change_d, reg_ml, small_p.

The Kaiser–Meyer–Olkin (KMO) value was 0.765, and the Bartlett spherical test also showed a significance probability of less than 0.05, indicating that the factor analysis model was suitable. The results of factor analysis of FCB are shown in Table 2.

Based on the contents of each factor, they were named as follows: Factor 1, Healthy; Factor 2, Cost-effective; Factor 3, Soundhabit; Factor4, Convenience. All factor loadings were above 0.4, satisfying the validity of the overall factors. The analysis was conducted without exclusion or adjustment of additional items.

Reliability analysis was performed to verify the internal consistency of the sub-factors of FCBs. In general, Cronbach’s α is judged to be reliable above 0.7, but Mueller and Hancock (2019) argued that even if it reaches 0.5 or higher it could be reliable. As a result of calculating Cronbach’s α for FCBs, as shown in Table 3, all were above 0.5, indicating that the reliability of the main variables of this study was acceptable.

#### 3.5.2. USM

Social media usage frequency was measured on a scale of 1 to 3: 1 = not at all; 2 = sometimes; 3 = often. In this study, we classified 2 and 3 as the ‘user’ group, and 1 as the ‘non-user’ group to confirm if there were any significant differences in FCB between the two groups according to the use of social media.

#### 3.5.3. Dietary Satisfaction

For measuring dietary satisfaction, the question “How satisfied are you with your current dietary lifestyle” was asked. A 5-point Likert scale was used for answers: 1 (very unsatisfied), 2 (unsatisfied), 3 (neutral), 4 (satisfied) and 5 (very satisfied).

### 3.6. t-Test

The *t*-test is the most common statistical method to verify whether the difference between the means of two groups is significant. The following three assumptions are made: independence, normality, and equivalent variance. The mean of the normal distribution is measured based on the Student’s t-distribution. This study conducted independent sample *t*-tests. The difference of the means in each FCB depending on USM was compared.

### 3.7. Analytic Model for SEM

This study also used SEM for examining the correlation between FCB and dietary satisfaction mediated by USM. All analyses were performed using Stata version 16.1 (Stata Corp, College Station, TX, USA). Statistical significance was set at *p* < 0.05. Equations (1)–(4) were used for setting the latent variables: Healthy, Costeffective, Soundhabit and Convenience. The structural model is presented with the following Equations (5)–(12):

(1) $$Healthy=\beta_{0}+\beta_{1}{\mathrm{cal\_nut}}_{i}+\beta_{2}{\mathrm{health\_f}}_{i}+\beta_{3}{\mathrm{safety\_f}}_{i}+\varepsilon_{i}$$

(2) $$Costeffective=\beta_{0}+\beta_{4}{\mathrm{price\_c}}_{i}+\beta_{5}{\mathrm{quality\_c}}_{i}+\varepsilon_{i}$$

(3) $$Soundhabit=\beta_{0}++\beta_{6}{\mathrm{taste\_f}}_{i}+\beta_{7}{\mathrm{n\_food}}_{i}+\beta_{8}{\mathrm{reject\_h}}_{i}+\varepsilon_{i}$$

(4) $$Convenience=\beta_{0}+\beta_{9}{\mathrm{simple\_b}}_{i}+\beta_{10}{\mathrm{hmr\_d}}_{i}+\varepsilon_{i}$$

(5) $$Healthy=\beta_{0}+\beta_{1}sns_{i}+\varepsilon_{i}$$

(6) $$Costeffective=\beta_{0}+\beta_{2}sns_{i}+\varepsilon_{i}$$

(7) $$Soundhabit=\beta_{0}+\beta_{3}sns_{i}+\varepsilon_{i}$$

(8) $$Convenience=\beta_{0}+\beta_{4}sns_{i}+\varepsilon_{i}$$

(9) $$\mathrm{satisfy\_dl}=\beta_{0}+\beta_{1}sns_{i}+\beta_{1}Healthy_{i}+\varepsilon_{i}$$

(10) $$\mathrm{satisfy\_dl}=\beta_{0}+\beta_{2}sns_{i}+\beta_{2}Costeffective_{i}+\varepsilon_{i}$$

(11) $$\mathrm{satisfy\_dl}=\beta_{0}+\beta_{3}sns_{i}+\beta_{3}Soundhabit_{i}+\varepsilon_{i}$$

(12) $$\mathrm{satisfy\_dl}=\beta_{0}+\beta_{4}sns_{i}+\beta_{4}Convenience_{i}+\varepsilon_{i}$$

## 4. Results

### 4.1. Descriptive Stastical Analysis

The results for the descriptive statistics of the variables used in this study are shown in Table 4. The absolute value of skewness was distributed between 0.055 and 0.493 (−2.00 < skewness < 2.00), and the absolute value of kurtosis was distributed between 2.507 and 2.936 (−4.00 < kurtosis < 4.00). Accordingly, these analytic results matched the conditions required for the normal distribution.

### 4.2. FCB Depending on Social Media Usage

The difference of means between variables in FCBs by USM was examined for each group using *t*-tests. As a result of testing whether this difference was significant, variables where there was a difference in the FCBs of adolescents depending on their USM were as follows: n_food (t = 2.134, *p* < 0.05), reject_h (t = 3.513, *p* < 0.001), simple_b (t = −3.893, *p* < 0.001), hmr_d (t = −3.245, *p* < 0.01). On the other hand, the following values did not show a significant difference depending on USM: cal_nut, health_f, safety_f, price_c, quality_c, taste_f. The results of the *t*-tests are summarized in Table 5.

### 4.3. Relationship between FCB and Dietary Satisfaction Mediated by USM

The goodness of fit in SEM could be confirmed using Baron and Kenny’s evaluation of mediating effects. The validity of the questionnaire used in the survey (n = 622) was confirmed through confirmatory factor analysis (CFA). The fit of the overall effect model was as follows: the root mean squared error of approximation (RMSEA) was 0.074, which was lower than 0.080; accordingly, it indicated a moderate fit [55]; the comparative fit index (CFI) was 0.894 and the Tucker–Lewis index (TLI) was 0.857, which, being close to 0.90, was interpreted as an acceptable fit [56,57]. The CFA results demonstrated a favorable fitness of data in the model.

Table 6 presents the results of the analysis of direct, indirect, and total effect for examining each path that affects dietary satisfaction based on SEM. There was no direct relation between USM and dietary satisfaction, and only the indirect effect of USM mediated by the FCB of preferring convenience was statistically significant. Accordingly, the FCB of preferring convenience had a full negative mediation effect on the relationship between USM and dietary satisfaction.

## 5. Discussion

Hoffman and Novak [24] found a positive association between social media goal pursuit and the happiness of content creators and connecters. However, the results of this study were inconsistent with their study. This study revealed a negative relationship between USM and dietary satisfaction in the food sector. There is consensus that the sharing of social media content itself can produce perceived pleasure [58]. In particular, adolescent consumers form a para-social bond through purchasing behavior [59]. Various factors, including sensory factors, service satisfaction, eating habits, and food safety are involved in evaluating dietary satisfaction, and although FCBs vary with USM, the following results should be considered: USM had no direct effect on dietary satisfaction and there were only indirect effects mediated by FCB.

USM mediated by the FCB of preferring convenience decreased dietary satisfaction. In terms of the results, there have been many studies showing that USM affects adolescents’ intake of convenience food such as fast food [6,7]. However, the findings from this study suggest that the effects of adolescents’ USM on their dietary lifestyle lower their dietary satisfaction. Governments, parents, food companies, and adolescents themselves needs to pay attention to the fact that the tendency of adolescents to prefer convenience ultimately decreased their dietary satisfaction. According to a study by Olsen and Tuu [60] on Vietnamese teenagers, considering future effects and healthy eating values were factors that affected convenience food consumption negatively. The FCB of seeking convenience is still considered unhealthy for adolescents.

USM mediated by the FCB of considering health did not affect dietary satisfaction. It is thought that food consumption while considering health will increase dietary satisfaction regardless of age [36,37]. However, this relationship could not be confirmed in this study on adolescents’ FCB. Health-related social media activities such as searching related information or sharing content have been thought to be a main trend in recent years, but this did not have any effect on dietary lifestyle. Adolescents’ food-related social media activity might be performed with a different motive or purpose, rather than being a direct influencing factor on their dietary lifestyle.

USM mediated by the FCB of valuing cost-effective consumption did not affect dietary satisfaction. In the era of mass production of various kinds, consumers who have more options due to fierce competition have been increasing their consumption in considering cost-effectiveness [42]. Food consumption is also showing undeniable patterns of cost-effectiveness being considered. However, adolescents, whose consumer sovereignty is determined by their parents, seem to be less affected by these recent consumption trends.

USM mediated by the FCB of keeping sound eating habits did not affect dietary satisfaction. Education authorities and parents want youths to have sound eating habits, and they educate them in that direction [44,45,46,47]. However, it is judged that adolescents who use social media are not interested in maintaining a sound eating habit, or do not realize the importance of it as much as their parents’ generation.

## 6. Conclusions

A countermeasure that mediates between the growing dependence of adolescents on social media and the increasing trend in the FCB of preferring convenience is needed. The leaders of the food industry should present a guideline to identify information on convenience food that adolescents access easily. In addition, the government should strengthen regulations on convenience foods targeted towards adolescents. In particular, it is necessary to increase the accuracy of product information by regulating online advertisements, and to eradicate exaggerated advertisement. Governments could consider increasing parental involvement to establish regulating policies [61]. Food companies that supply food to adolescents need to establish an approach that can reverse the current situation in which dietary satisfaction declines as adolescents consume more convenience food. Food companies also need to improve the quality of convenience food rapidly to improve dietary lifestyles.

There might be something among adolescents’ FCBs that acts as a positive factor in increasing dietary satisfaction. A clue can be found in their recent consumption trends. They are regarded as the mainstream of value consumption [62]. They are oriented towards eco-friendliness [63,64]. They choose food sharing, one of the representative habits of eco-friendly consumption. In fact, food sharing itself does not lead to food waste reduction, but pursuing it creates expectations for environmental and economic benefits at the consumer level [65]. In particular, their adoption of food sharing is expected to make it possible to increase dietary satisfaction through more effective food waste disposal [66].

Although there have been many existing studies reporting that adolescent USM has a positive or negative effect on their dietary lifestyle, ours is a rare study on the mechanisms, to verify a meaningful relationship between adolescents’ USM and their dietary lifestyle. This study contributes to filling a gap in the literature by verifying the relationship between adolescents’ USM, their FCBs and dietary satisfaction, by studying national statistics. This study could be used as a reference for educational institutions in charge of school meals, or nutrition education for adolescents, to set guidelines for increasing the dietary satisfaction of adolescents. Additionally, food companies that provide food for adolescent could gain insights from this study to help them fundamentally improve their business.

Nevertheless, this study has the following limitations. This study extracted variables of interest from public open data, which have already been investigated. Therefore, further investigation and analysis of factors related to adolescents’ FCBs were not possible. In the same context, the FCBs derived from them might not be representative of the FCBs of adolescents. The main target of the original questionnaire was to examine adults’ FCBs. The sample of adolescents is smaller than that of adults. Furthermore, the reliability test result for the FCB factor of keeping sound eating habits was lower than 0.6, which is the normal standard of reliability. The data were collected up to 2020, and were therefore affected by the special circumstances of 2020.

In future studies, we will conduct advanced research on adolescents’ FCBs in a more multifaceted manner, and use an improved questionnaire reflecting new trends. In addition, we need to increase the sample of adolescent respondents and use time series data. This study should secure the reliability of 0.6 or higher by excluding the items that were lower.

## Figures and Tables

**Table 1:** Operational definition of variables.

Category	Variables		Definition
explanatory variable (in analysis 1 and 2)	sns		usage of social media
dependent variable (in analysis 1)/mediating variable (in analysis 2)	Healthy	cal_nut	tendency to eat food considering calories and nutrients
		health_f	tendency to eat carefully selected food for one’s own health
		safety_f	tendency to choose food by considering safety rather than price or taste
	Costeffective	price_c	tendency to compare prices from several companies for the same product
		quality_c	tendency to check the quality level of food compared to the price before purchasing
		buy_cert	tendency to purchase HACCP- or GAP-certified products
	Soundhabit	change_d	tendency to change diet often for a variety of flavors
		taste_f	tendency to choose food based on taste
		n_food	tendency to try new types of food
		reg_ml	tendency to eat breakfast and every meal regularly
		reject_h	tendency not to eat foods with harmful risks such as suspected spoilage
	Convenience	simple_b	tendency to eat bread or fruit for a simple breakfast
		hmr_d	tendency to often use HMR or eating out/delivered food
		small_p	tendency to purchase small packaged foods or pre-processed agricultural products
dependent variable (in analysis 2)	satisfy_dl		satisfaction with dietary lifestyle

**Table 2:** Factor analysis of FCB.

	Factor 1	Factor 2	Factor 3	Factor 4
cal_nut	0.718	0.014	−0.113	0.107
health_f	0.745	−0.066	0.019	0.005
safety_f	0.547	0.175	0.133	−0.095
price_c	0.256	0.499	0.015	0.067
quality_c	0.344	0.500	−0.052	−0.001
taste_f	−0.079	−0.059	0.627	0.181
n_food	0.013	0.081	0.551	−0.016
reject_h	0.091	−0.060	0.425	−0.139
simple_b	0.097	−0.021	0.003	0.581
hmr_d	−0.020	0.064	0.139	0.571
Eigenvalue	2.627	0.828	0.601	0.216
Proportion variance	2.305	1.632	1.161	1.068
Cumulative variance	2.305	3.937	5.098	6.166
KMO = 0.765, Bartlett’s χ^2^ = 1546.45 (*p* < 0.001)				

**Table 3:** Reliability analysis.

Classification	Variables	Cronbach’s α
Healthy	cal_nut health_f safety_f	0.762
Costeffective	price_c quality_c	0.741
Soundhabit	taste_f n_food reject_h	0.575
Convenience	simple_b hmr_d	0.632

**Table 4:** Results of descriptive statistics.

Variables	Mean	S.D	Skewness	Kurtosis
cal_nut	3.260	0.873	−0.196	2.551
health_f	3.175	0.843	−0.162	2.986
safety_f	3.245	0.846	−0.283	2.873
price_c	3.229	0.838	−0.239	2.947
quality_c	3.136	0.829	−0.055	2.839
taste_f	3.779	0.772	−0.165	2.704
n_food	3.680	0.783	−0.256	2.918
reject_h	3.811	0.821	−0.321	2.778
simple_b	3.175	0.969	−0.493	2.507
hmr_d	3.286	0.943	−0.159	2.666

**Table 5:** Difference in FCBs between non-users and users of social media.

Variables	Non-User (n = 42)		User (n = 580)		t
	Mean	SD	Mean	SD	
cal_nut	3.40	1.03	3.25	0.86	1.109
health_f	3.26	0.79	3.16	0.84	0.689
safety_f	3.38	0.69	3.23	0.85	1.069
price_c	3.11	0.91	3.23	0.82	−0.887
quality_c	3.09	0.82	3.13	0.83	−0.334
taste_f	3.73	0.66	3.78	0.78	−0.361
n_food	3.92	0.77	3.66	0.78	2.134 *
reject_h	4.23	0.75	3.78	0.81	3.513 ***
simple_b	2.61	0.88	3.21	0.96	−3.893 ***
hmr_d	2.83	1.12	3.31	0.92	−3.245 **

* *p* < 0.05, ** *p* < 0.01, *** *p* < 0.001.

**Table 6:** Analytic result of effect.

Path	Coefficient	95% CI	
		Lower	Upper
USM → Dietary satisfaction	−0.054	−0.135	0.026
USM → Healthy → Dietary satisfaction	−0.006	−0.031	0.008
USM → Costeffective → Dietary satisfaction	0.002	−0.015	0.024
USM → Soundhabit → Dietary satisfaction	0.001	−0.002	0.020
USM → Convenience → Dietary satisfaction	−0.027 **	−0.069	−0.003

** *p* < 0.01, CI: Confidence Interval.

## Data Availability

The data used in this study are available to other authors who require access to this material.
